# The association of complex genetic background with the prognosis of acute leukemia with ambiguous lineage

**DOI:** 10.1038/s41598-021-03709-7

**Published:** 2021-12-21

**Authors:** Jin Huang, Jing Zhou, Min Xiao, Xia Mao, Li Zhu, Songya Liu, Qinlu Li, Jin Wang, Jianfeng Zhou, Haodong Cai, Gaoxiang Wang

**Affiliations:** Department of Hematology, Tongji Hospital, Tongji Medical College, Huazhong University of Science and Technology, 1095# Jiefang Ave., Wuhan, 430030 People’s Republic of China

**Keywords:** Cancer, Genetics

## Abstract

Acute leukemia with ambiguous lineage (ALAL) is a rare and highly aggressive malignancy with limited molecular characterization and therapeutic recommendations. In this study, we retrospectively analyzed 1635 acute leukemia cases in our center from January 2012 to June 2018. The diagnose of ALAL was based on either EGIL or 2016 WHO criteria, a total of 39 patients were included. Four patients diagnosed as acute undifferentiated leukemia (AUL) by both classification systems. Among the patients underwent high-throughput sequencing, 89.5% were detected at least one mutation and the median number of gene mutation was 3 (0–8) per sample. The most frequently mutated genes were NRAS (4, 21%), CEBPA (4, 21%), JAK3 (3, 16%), RUNX1 (3, 16%). The mutations detected in mixed-phenotype acute leukemia (MPAL) enriched in genes related to genomic stability and transcriptional regulation; while AUL cases frequently mutated in genes involved in signaling pathway. The survival analysis strongly suggested that mutation burden may play important roles to predict the clinical outcomes of ALAL. In addition, the patients excluded by WHO criteria had even worse clinical outcome than those included. The association of the genetic complexity of blast cells with the clinical outcomes and rationality of the diagnostic criteria of WHO system need to be evaluated by more large-scale prospective clinical studies.

## Introduction

Majority of acute leukemia (AL) can be accurately diagnosed and classified into acute myeloid leukemia (AML) or acute lymphoid leukemia (ALL) according to morphologic, cytochemical, and immunologic features^1^. However, less than 5% of patients can’t be clearly assigned to the well-established subsets of AL as lacking expression of lineage-specific markers, or presenting mixed immune phenotype more than one lineage. In the past two decades, the diagnostic criteria for such kinds of leukemia were controversial, and the nomenclature was constantly improving. The European Group for the immunological characterization of leukemia (EGIL) separately defines them as acute undifferentiated leukemia (AUL), which means no lineage related antigens are expressed, and bi-phenotypic acute leukemia (BAL) when either two separate blast populations are encountered or a single blast population demonstrating evidence of both myeloid and lymphoid differentiation concurrently^2^. WHO (2008/2016) classification system collectively groups these rare disorders as acute leukemia with ambiguous lineage (ALAL), which may possibly arise from hematopoietic pluripotent stem cells^3^. The new classification system proposes the diagnosis of mixed-phenotype acute leukemia (MPAL) and emphasizes the impact of CD19 for B-cell lineage, CD3/cCD3 for T-cell lineage, and cytoplasmatic myeloperoxidase (MPO) for myeloid lineage, respectively^4,5^. The diagnosis of ALAL largely depends on the immunophenotype, and cases with AML specific recurrent cytogenetic abnormalities are classified to AML as defined in WHO classification system. Based on the markers expressed on the blast cells, BAL/MPAL can be further divided in to B/M, T/M, B/T, and B/T/M subtypes.

It has been widely recognized that the clinical outcome of ALAL is markedly adverse, and the overall survival (OS) ranges from 9 months to 3.5 years according to published data^6–8^. Poor response to conventional chemotherapy and high rate of relapse may partly explain the unsatisfactory clinical outcome. Some studies reported ALL-like therapy might be superior to AML-based protocols, and long-term survival can only be improved by allogeneic stem cell transplantation^8–11^. However, till now, no consistent treatment strategies are recommended and overall survival is 20–40% for adults and 47–75% for pediatric ALAL patients^10,12,13^. Another important issue which can’t be ignored is that the new WHO diagnostic system, compared with the previous EGIL criteria, eliminates a certain body of patients from ALAL. These patients may be diagnosed as AML or ALL, and receive the chemotherapy accordingly. Whether their diagnosis and treatments are appropriate needs more comprehensively evaluation.

Better characterizing this subset of leukemia will shed new light in overcoming this special entity of disease. However, no distinctive biological characteristics have been identified. As previous studies documented, cytogenetic abnormalities were detected in 59–91% of patients with MPAL and 80–90% of patients with AUL. Recently, Alexander et al. reported the sequencing data of 159 pediatric patients with ALAL, and showed rearrangement of ZNF384 were appeared in nearly half of B/M MPAL^14,15^. With the progressions in molecular genetics, high-throughput sequencing technology makes it possible to investigate this kind of disease more profoundly in molecular level^6,15,16^. Several studies demonstrated the genetic landscape of MPAL and showed patients clustered to either AML-like or ALL-like MPAL based on methylation profiling could be benefited from corresponding therapies^15,16^. However, the correlation between the genetic mutation and the clinical outcomes of ALAL was not fully elucidated.

In this study, we retrospectively analyzed the patients with de novo acute leukemia from January 2012 to June 2018 in Tongji Hospital, Tongji Medical Collage of Huazhong University of Science and Technology and aimed to examine a wide spectrum of gene mutations in patients with ALAL with defined under WHO or EGIL criteria, to determine their clinical relevance with clinical outcomes of such dire leukemia and the clinical applicability of these two diagnostic systems.

## Results

### Basic characteristics of patients enrolled in this study

In all 1635 patients with acute leukemia admitted to our center over a period of more than five years, 39 patients (2.4%) satisfied the criteria of EGIL, and 30 (1.8%) patients were diagnosed as AUL or MPAL according to the WHO standards. For all 39 patients, mean age was 39 years and 23 (59%) of them were male. The median value of WBC count on presentation was 11 × 10^9^, hemoglobin 80 g/L, platelet 71 × 10^9^, and bone marrow blasts 80% (Table 1). There was no significant difference regarding to the baseline and clinical characteristics between the patients included (WHO 2016, n = 30) and excluded (EGIL-WHO, n = 9) by WHO criteria, except that patients excluded by WHO system had a lower level of platelet count (p = 0.039) and did not express MPO (p = 0.001). According to the FAB criteria, 14 (35.9%) patients were classified as ALL, and 5 (12.8%) patients displayed an AML morphology, and others were classified as AUL (7.7%), mix phenotype leukemia (2.6%), or inclusive (41%) (Supplementary Table 1). The clinical diagnosis of ALAL can hardly be realized by FAB diagnosis system.

**Table 1:** Clinical and laboratory features of patients in this study.

Characteristics	EGIL	WHO 2016	EGIL-WHO	p value*
	(n = 39)	(n = 30)	(n = 9)	
Sex, m/f	23/16	18/12	5/4	0.812
Age, y	39 ± 16.6	39 ± 14.9	38 ± 22.4	0.935
WBC, × 10^9^/L	11 (3–40)	10 (3–39)	37 (2–107)	0.588
HB, g/L	80 (62–106)	86 (64–105)	68 (42–124)	0.501
PLT, × 10^9^/L	71 (35–131)	79 (44–145)	29 (7–80)	0.039
BM blast (%)	80 (55–92)	76 (51–90)	92 (75–97)	0.069
**Karyotype abnormality, N (%)**				
Abnormal	17 (53.1)	15 (60.0)	2 (28.6)	0.141
Ph+	7 (21.9)	7 (28.0)	0 (0)	0.113
MLL rearrangement	1 (3.1)	0 (0)	1 (14.3)	0.055
Complex	9 (28.1)	8 (32.0)	1 (14.3)	0.357
**Immunophenotype profile, N/tested**				
CD34+	36/39	27/30	9/9	0.323
MPO+	19/39	19/30	0/9	0.001
CD13+	30/38	22/29	8/9	0.402
CD11c+	9/17	7/13	2/4	0.893
CD14+	5/29	3/21	2/8	0.495
CD64	6/25	5/17	1/8	0.356
CD33+	24/34	17/25	7/9	0.581
CD117+	24/36	16/27	8/9	0.102
CD19+	17/38	15/29	2/9	0.120
CD20+	4/23	4/19	0/4	0.313
CD22+	10/13	8/10	2/3	0.631
CD10+	18/32	15/24	3/8	0.217
CD79a+	17/36	14/27	3/9	0.335
CD3+/cCD3+	20/37	14/28	6/9	0.383
CD2+	9/23	6/16	3/7	0.809
CD5+	11/24	9/16	2/8	0.148
CD7+	25/38	18/29	7/9	0.386
Patients with mutations, N/tested	17/19	15/17	2/2	0.608

*EGIL* European Group for the Classification of Acute Leukemia, *WHO* World Health Organization, *BM* bone marrow.

*p values were evaluated by comparing EGIL-WHO with WHO 2016.

Four AUL cases (10%) expressed no lineage specific markers but CD34, HLA-DR with or without TdT. Among the remaining cases, 16 (41%) cases had a myeloid and B-lymphoid phenotype (B-M), 16 (41%) for a myeloid and T-lymphoid phenotype (T-M), 2 (5.1%) for a B-T-lymphoid phenotype (B-T), and 1 cases (2.6%) with evidence of tri-lineage concomitant expression (myeloid, B, and T lymphoid [B-T-M]; Supplementary Table 1).

Among all, 32 patients underwent karyotyping, and 17 (53.1%) of them were abnormal. Complex karyotypes were observed in 9 (28.1%) patients with a high degree of heterogeneity as shown in Table 2. The most frequent abnormal karyotype is t (9; 22) (q34; q11.2), resulting in the BCR-ABL1 fusion gene, appeared in 7 patients. Interestingly, 6 patients were B-M MPAL while one of them was T-M MPAL. MLL rearrangement was observed in a patient diagnosed as T-M MPAL (Table 2). In addition to BCR-ABL and SET/CAN, which were previously reported, we also detected E2A-PBX1 fusion gene in a B-M MPAL patient, in which two lineage markers were detected in one cluster of blast (Table 2).

**Table 2:** Detailed information of the patients with cytogenetic abnormalities.

Patient no	Gender	Age (years)	EGIL classification	2016 WHO classification	Gene fusion	Chromosome karyotype	CR	OS (day)
1	Male	24	BAL	MPAL, B-M, NOS	E2A/PBX1	46, XY, [9]/43, XY,b (1q), 6q-, -9, -10,11q-,-15,-17,-18,19p-,-21 [1]	Yes	478+
2	Female	42	BAL	MPAL, BCR/ABL+	BCR/ABL (P210)	46, XX, t(9;22) (q34;q11) [20]	Yes	115+
3	Female	24	BAL	MPAL, B-M, NOS	Neg	46, XX, t (2:5) (p21:p15), del (6) (q16-q22), t (9:x) (p24:q21)	Yes	578
13	Female	56	BAL	MPAL, T-B, NOS	Neg	46, XY, dic(11:22)(q24;q12),del(12)(q22) [7]/46,xx [3]	No	381
15	Male	20	BAL	MPAL, BCR/ABL+	BCR/ABL (P190)	46, XY, r (5) (p15q35), t (9; 22) (q34, q11) [14]/46, XY, t (9; 22) (q34; q11) [6]	Yes	530+
16	Female	58	BAL	MPAL, T-M, NOS	SET/CAN	Neg	No	360
19	Male	26	BAL	MPAL, T-B, NOS	Neg	Subtriploid karyotype	Yes	602+
21	Female	29	BAL	MPAL, BCR/ABL+	BCR/ABL	46, XX [6]/[CP] 46, XX, 2q+, t (9; 22) (q34; q11), 14p+ [8]	Yes	579+
22	Male	25	BAL	T-ALL	–	43–47, XY, t (4; 11) (q21; p15), +6, del (6) (q21), del (7), (q21), del (7) (q14), add (9) (p24), add (11) (p15), add (14) (q32), -17, del (17), (p13), +ace [cp6]/46, XY, [14]	No	83
25	Female	43	BAL	MPAL, T-M, NOS	–	47, XX, +21 [4]/46, XX [2]	Yes	545+
26	Female	41	BAL	MPAL, BCR/ABL+	BCR/ABL (P210)	–	Yes	2293+
29	Female	60	BAL	B-ALL	Neg	47, XX, t(8; 13), (q10; p10), +22 [10]	No	51
31	Male	50	BAL	MPAL, BCR/ABL+	BCR/ABL (P210)	47, XX, +11 [9]/46, XX, [1]	Yes	850+
32	Female	36	AUL	AUL	Neg	47, XX, +8 [3]	No	382
36	Male	18	BAL	MPAL, BCR/ABL+	BCR/ABL (P190)	Neg	Yes	565+
37	Male	42	BAL	MPAL, BCR/ABL+	BCR/ABL (P190)	49, XY, t (9; 22) (q34; q11), +10, +21, +der (22) t (9; 22) (q34; q11) [1]/53, idem, M31 + X, +1, +5, +8 [1]/46, xy [18]	No	731
44	Male	44	BAL	MPAL, T-M, NOS	Neg	46, XY [6]/47, XY, +10 [14]	Yes	351+
45	Male	23	AUL	AUL	Neg	47, XY, add (2) (q37), +4, t (10;11) (p12;q21), -17, +mar [4]/47, idem, add (2) (q37) [1] /46, XY, [15]	Yes	141

### Genetic testing

Genetic testing was conducted in 27 cases. Nineteen out of them were tested by 173-gene panel (Supplementary Table 2) in next-generation deep sequencing platform, and 8 of them, who admitted in hospital in earlier time, were tested by Sanger sequencing. 17 cases (89.5%) were detected at least one mutation by high-throughput sequencing system, while only 2 patients were positive mutated by screening limited hot spot mutation of AML by Sanger sequencing. The two mutated genes detected by first-generation sequencing were the CEBPA in No. 36 patient and the FLT3-ITD repetitive tandem sequence in No. 42 patient, respectively.

Based on the data of the 19 patients who had been sequenced in the next-generation platform, 53 high-confidence mutations were detected in 33 genes in 17 patients. The two patients with no detectable mutations were both B-M phenotype. The median number of gene mutation was 3 (0–8) per sample. The most frequently mutated genes were NRAS (4, 21%), CEBPA (4, 21%), JAK3 (3, 16%), RUNX1 (3, 16%). Mutations in genes such as DNMT3A, ETV6, IDH2, KMT2D, KRAS, NOTCH1, PHF6, TP53, and WT1 were detected in two different patients. CEBPA mutations were detected in 3 patients with M-T phenotype and one with B-T phenotype. Notably, the mutations in NRAS gene (reoccurring in 4 cases) were concentrated at its 12th/13th amino acid site.

**Figure 1:**
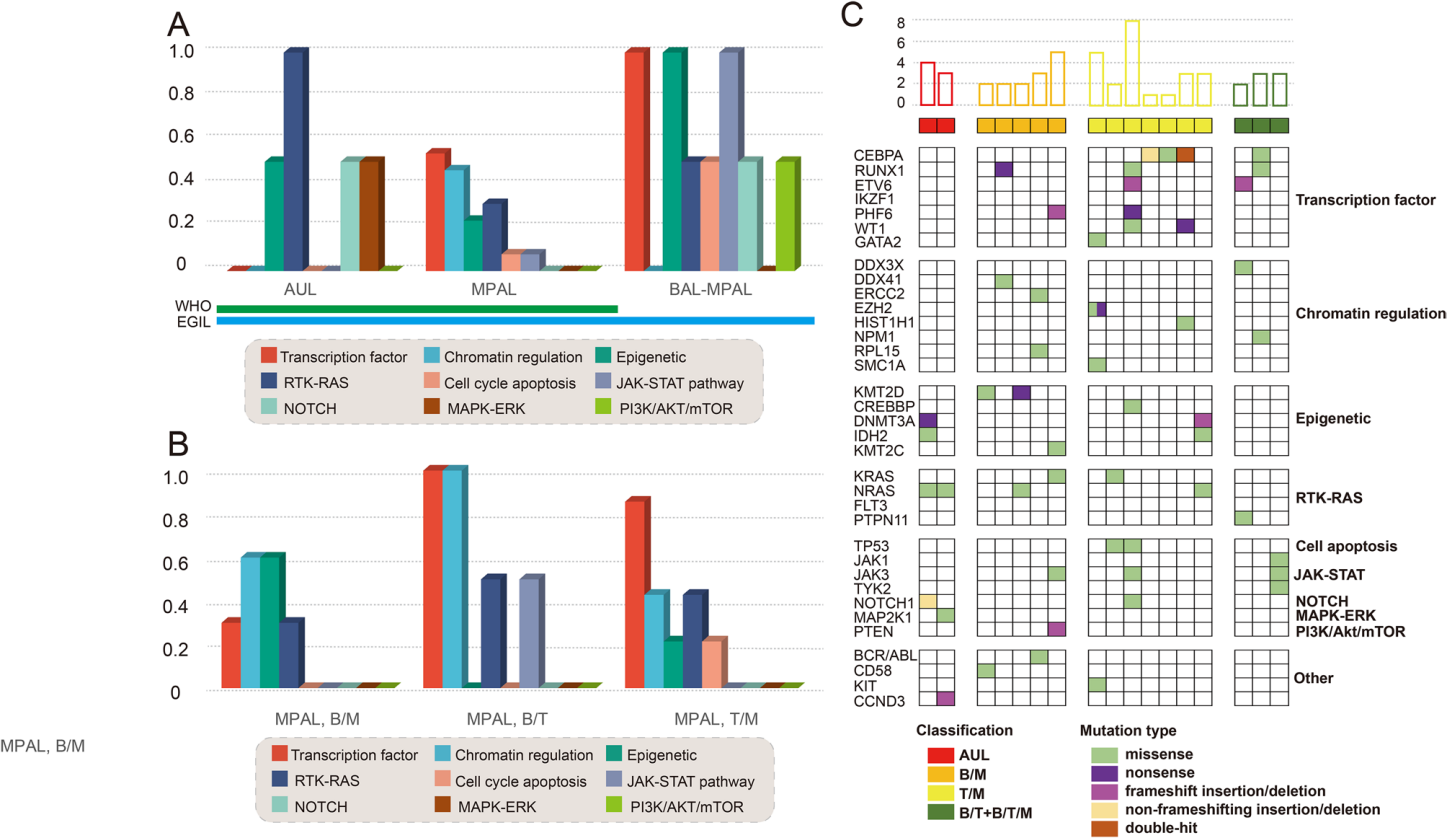
Landscape of somatic mutations in ALAL by next-generation sequencing. (**A**) Landscape of mutations detected in acute undifferentiated leukemia (AUL, n = 4), mixed-phenotype acute leukemia (MPAL, n = 13) and cases who were satisfied EGIL but excluded by WHO (n = 2). (**B**) Landscape of mutations detected in MPAL, including myeloid/B-lymphoid phenotype (MPAL B/M, n = 4), B-lymphoid/T-lymphoid (MPAL B/T, n = 3), and myeloid/T-lymphoid phenotype (MPAL T/M, n = 6). Y-axis represents the ratio of patients with mutations and the patients performed next-generation genetic testing. Columns with different colors represent genes belong to different categories according their attributes. (**C**) Detailed information of the 17 cases who detected mutations. Each column represents a case and each raw represents a gene. The top bar graph shows the number of mutations detected in each sample. The row directly underneath that graph show the immunophenotypes and Different colors of squares represent various mutation type.

We divided genes into the following categories according to diagnostic system and gene attribution such as transcription factor, chromatin regulation, epigenetics, cell apoptosis, signaling pathway (RTK-RAS, NOTCH, MAPK-ERK, PI3K/AKT, JAK-STAT), and others. Fifteen patients (2 AUL and 13 MPAL) satisfied both WHO and EGIL criteria, and two patients satisfied the EGIL criteria, but excluded by WHO classification system. The results showed that mutations detected in MPAL cases enriched in transcription factor, chromatin regulation and epigenetic, all related to genomic stability and transcriptional regulation; while AUL cases frequently mutated in genes in signal pathway regulation such as RTK-RAS, NOTCH, and PI3K/AKT (Fig. 1A). The mutation profile of the 2 patients excluded by WHO diagnostic criteria were analyzed separately, and the limited data suggested that the genetic background of these cases were even more complex than those included patients (Fig. 1A). There was no significant difference in the distribution of B/M, B/T, and T/M gene mutations (Fig. 1B). The detailed information profile was shown in Fig. 1C.

### Survival analyses

Survival analyses were used to compare the prognosis of clinical subsets and types of treatment. Up to January 16, 2019 (mean follow up time 428 days), 34 patients received chemotherapy, while others, according to patients' wishes or limitation of general condition, only received symptomatic support treatment. Among the patients who received chemotherapy, 7 cases received ALL-like therapy, 6 cases received AML-like therapy, and 21 cases received AML + ALL combined therapy. Decitabine was included in the therapy of 9 patients, 5 patients received tyrosine kinase inhibitors (TKI), and 4 patients received allogeneic hematopoietic stem cell transplantation (HSCT).

**Figure 2:**
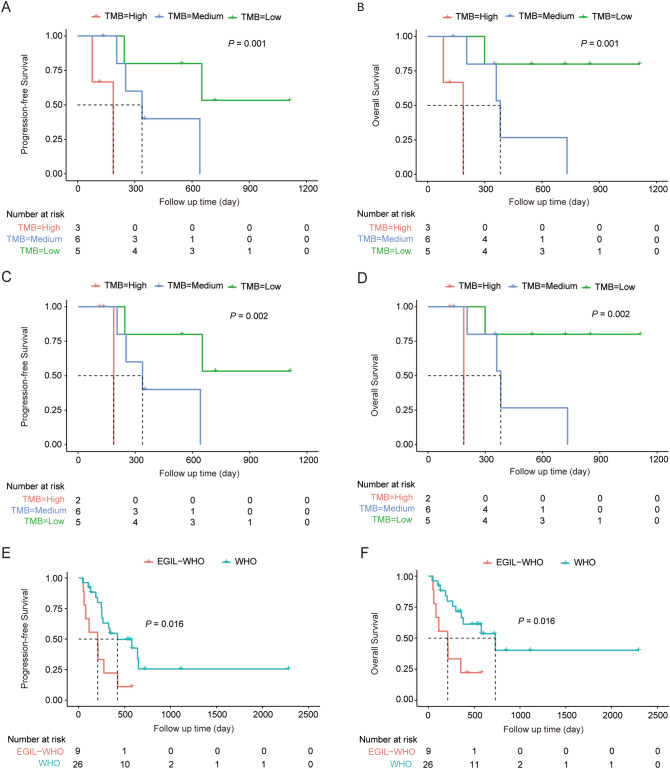
Outcomes of patients with ALAL by different clinical subsets. (**A**,**B**) Kaplan–Meier curve of PFS (**A**) and OS (**B**) stratified by tumor mutation burden/person by EGIL criteria. (**C**,**D**) Kaplan–Meier curve of PFS (**C**) and OS (**D**) stratified by tumor mutation burden/person by WHO 2016 criteria. (**E**,**F**) Kaplan–Meier curve of PFS (**C**) and OS (**D**) stratified by different classification systems (green curves indicating patients included in WHO 2016 criteria and red curves indicating patients who were included by the EGIL classification but excluded by the WHO 2016 classification).

Among all the 35 BAL/MPAL patients, 20 patients (57.1%) achieved complete remission (CR) at least once. The CR rate was not affected by immunophenotype (p = 0.163), complex karyotype (p = 0.833), whether mutated (p = 0.208), or chemotherapy regimen (p = 0.410); while showed a significant association with mutation complexity (p trend = 0.006) (Supplementary Table 3). During the follow up, 23 cases (65.7%) progressed or died. Conventional risk factors including age, WBC counts, complex cytogenetics, and therapy were evaluated among different subgroups discriminated by gene mutation burden, and the results showed that all the factors were balanced distributed (Supplementary Table 4). Of all factors considered, mutation complexity, calculated by (aberrant CNV number + mutation number)/panel size (Mb), was significantly associated with PFS and OS (Log rank p = 0.001 and Log rank p = 0.001 respectively) (Fig. 2A,B and Supplementary Table 5). When the WHO diagnostic criteria were applied, we obtained the similar results in the patients included. In specific, mutation complexity was the only clinical risk factor that remarkably associated with CR rate (p = 0.002), PFS (Log rank p = 0.002), and OS (Log rank p = 0.002), manifested as patients with more complex mutation profiles had much worse clinical outcomes (Fig. 2C,D and Supplementary Table 6). Of note, 9 patients were excluded according to WHO 2016 classification system. Eight of them were diagnosed as acute lymphoblastic leukemia, and one was determined as unclassified. We found the patients excluded by WHO criteria had an even worse prognosis than those patients included, characterized as shorter PFS (Log rank p = 0.016) and OS (Log rank p = 0.016) (Fig. 2E,F and Supplementary Table 5). Whether the clinical diagnosis and treatment of these subsets of patients is the optimal strategy may need to be further explored.

Refer to the 4 AUL cases, three patients received chemotherapy and only one patient who received AML + ALL therapy (25%) achieved complete remission (CR). The median progression-free survival (PFS) is 132 days, and the median overall survival (OS) is 262 days. Two of them conducted NGS, and the one with higher mutation burden had worse disease progression with PFS (51 days vs. 133 days) and OS (51 days vs. 382 days), respectively.

## Discussion

ALAL is a rare and aggressive entity with heterogeneous immunophenotypic, cytogenetic and molecular features. Our study provided the genetic landscape of ALAL by screening the hot-spots in 173 genes strongly associated myeloid and lymphoid malignancies and evaluated the clinical characteristics with the dismal clinical outcomes.

Recent studies also emphasized the important roles of somatic mutations in the diagnoses and treatment strategy of ALAL^6,15–17^. These genetic data and our study both revealed that MPAL shared mutations with both AML and ALL, which indicated it would probably originated from early hematopoietic stem cells. B/M phenotype and T/M phenotype shared genetic alterations in the category of transcription factor and epigenetics, which was consistent with our findings. However, we did not observe the tendency towards to signaling pathway mutations of T/M and B/M cases as previous reported^16^. Limited number of patients may be the key reason. In this study, we analyzed 4 patients with AUL and 15 acute leukemia with mix-phenotype (BAL/MPAL) by high throughput sequencing and the results demonstrated these two sub-types of ALAL possessed different mutation files. We anticipate that the genetic information revealed by high throughput sequencing will innovate the medical care of ALAL in the future.

Another important point of view of our study is the deep exploration of the diagnostic system of ALAL. EGIL classification guideline was first proposed in 1995 and adopted in WHO criteria in 2001^2^. Updated WHO 2008/2016 classification implicated significant modification to the EGIL score system and quite a number of patients were eliminated outside ALAL diagnosis, just as we found in our study^4,5^. This part of patients was diagnosed as AML or ALL, and received corresponding treatment. However, whether the remarkable change is suitable and beneficial for the patients is still controversial, and the conflict focuses on the part of patients excluded by the new WHO system^3,18^. In this study, we found the patients excluded by WHO system had an even worse prognosis (Log rank p = 0.016 for PFS and Log rank p = 0.016 for OS), which was consistent with a previous reported clinical study^19^. In spite of this, we also found that the patients, satisfied the EGIL criteria but excluded by WHO diagnose system, shared the similar basic characteristics and immunophenotype with the patients included as showed in Table 1. Of the 9 patients excluded by WHO from ALAL, 2 cases underwent high throughput sequencing, and the results showed both of them carried completed genetic mutations distributed in genes like PTEN, KRAS, JAK3, KMT2C, WT1, ETV6, TP53, JAK3, RUNX1, NOTCH1, PHF6, and CREBBP. One of them showed no response to conventional chemotherapy, and unfortunately died 83 days after the diagnose. This is the first study to investigate the genetic background of this part of patients, however, the number of patients is limited to describe the whole picture of these patients. As the diagnosis significantly dictates the therapeutic decision making, the clinical appliance of the WHO 2008/2016 criteria needs to be in more prospective clinical studies.

The role of genetic (cytogenetic and molecular) markers in guiding chemotherapy strategies as well as targeted therapy is inexperienced. According to the WHO2016 criteria, t (9; 22)/*P*h+ was considered as a separate entity. And the *P*h chromosome was revealed as the most common cytogenetic abnormality, with the incidence ranging from 17–41% in MPAL especially with B-M phenotype^10^. All *P*h+ patients should be confirmed as soon as possible due to the benefit probability from TKI^20,21^. In our study, we not only screened out 7 abnormal karyotyping patients with *P*h+ resulting in BCR/ABL fusion gene, but also further detected 1 case with T315I mutation. Therefore, it is evident for us to make a better choice on the utility of TKIs. New molecular information in combination with cytogenetic characteristics will be valuable for the identification of genetic changes in ALAL, and undoubtedly play an important role in precision medicine of ALAL.

IDH2 gene mutations frequently include R140Q and R172K^22^. Surprisingly among our 19 available samples for NGS, we found 2 cases (AUL: n = 1, MPAL, T/M, NOS: n = 1) harboring IDH2 R140Q mutation. Recently, novel selective inhibitors of IDH1/2 mutations, ivosidenib and enasidenib, have been approved for patients with relapsed or refractory IDH1/2-mutated AML^22^. There is a proof that a patient with IDH1-mutated AUL achieved molecular complete remission following ivosidenib^23^. This might be a promising step to open the door for targeted therapy in IDH-mutated ALAL^24^.

Interestingly, we first found E2A/PBX1 in MPAL, B/M, NOS, which has not been reported in ALAL before. To our knowledge, it is one of the most common translocations in pediatric B-ALL. In adult, although not exceptional, it appears approximately 3%^24^. The prognostic significance of E2A/PBX1 fusion gene in ALAL is acquired further research.

Given that the drugable genetic target is still limited, analyzing the aberrant or specific antigen expression on leukemia cells seems to be another potential strategy. We tried to detect some canonical surface markers which can be targeted by off-the-shelf chimeric antigen receptor T-cell (CAR-T) immunotherapy. Strikingly, we definitely found 11 patients (47.8% of tested, data not shown) expressed CD123 on the surface of the blast cells. It has been reported that CD123, as a more specific marker for AML cells, is expressed at low levels on normal hematopoietic stem/progenitor cells^25^. An CD123 antibody–drug conjugate IMGN632 had been approved by FDA for blastic plasmacytoid dendritic cell neoplasm (BPDCN), and CD123 CAR-T therapy exhibited a good effect in patients who are no longer responding to standard therapies^26–28^. It is potentially considered as a novel magic bullet for the therapeutics of ALAL with CD123 expression.

Some previous studies have explored the factors affecting the clinical prognosis of ALAL. From the results of meta-analyses with large sample size, significant survival benefit can be obtained from starting with ALL therapy followed by allogeneic hematopoietic stem cell transplantation irrespective of definition system^12,20,29–31^. The study of pediatric patients with ALAL also suggested that the ALL-like treatment scheme could benefit more. In this study, apart from immunophenotype, complex karyotype, treatment, mutation complexity is exclusively associated PFS and OS in ALAL, we observed the clue that the mutation burden in blast cells predicted higher risk of relapses and deaths. Due to the limitation of sample size, prospective studies with larger sample size will provide more powerful evidence.

Collectively, as the ALAL appears heterogeneous, it seems reasonable to establish stratification system of diagnosis and prognosis, especially based on the genetic features. Elucidating the genetic heterogeneity implicated in the process of leukemogenesis will provide insights into the pathogenesis and improve the management of the unusual subtype of acute leukemia.

## Methods

### Study design and participants

A retrospective analysis was performed based on data from 1635 de novo acute leukemia patients in Tongji Hospital (Wuhan, China) from January 2012 to June 2018. Standard procedures for diagnosis were performed, including morphology examination, multi-parameter flow cytometry analysis, molecular detection and karyotype analysis. According to EGIL or 2016 WHO criteria, 39 (2.4%) patients were enrolled in this study^1,4,5^. A total of 35 patients were designated as BAL according to EGIL scoring system and/or as MPAL if 2016 WHO criteria were fulfilled and 4 patients were diagnosed as AUL. The clinical characteristics, treatments and prognostic information were objectively collected from the medical records and validated by the physicians in charge or the telephone follow-up to the patients. AML-like therapy is defined as a “7 + 3” induction approach, followed with middle/high dose of Cytarabine for consolidation. ALL-like regimen refers to a standard treatment regimen of acute lymphoblastic leukemia with VDCLP as induction regimen and successive schedules such as hyper CVAD/MA as consolidation. AML + ALL like therapy is defined as a combination of ALL and AML drugs. AML + ALL-like are further divided into regimen based on AML (a schedule with “7 + 3” as induction followed with drugs in ALL treatment) and based on ALL (a schedule with DVCLP as induction followed with drugs in AML treatment). The detailed information of therapy were provided in Supplementary Table 1.

The study design was approved by the Ethics Committee of Tongji Hospital, Tongji Medical College, Huazhong University of Science and Technology according to the Declaration of Helsinki and all patients gave written informed consents to participate in this study.

### Targeted gene next-generation sequencing and mutation calling

Among the 19 patients underwent NGS, 3 patients carried gene sequencing reports when they were admitted to our hospital, the data of next-generation sequencing was obtained from their medical record. For the others, samples from 15 patients were sequenced in Ion Personal Genome Machine and 1 was sequenced in Illumina NextSeq 500/550 platform in our center. The panel designed for variants detection covered the main mutation hotspots of a total of 173 genes which were the most frequently detected in myeloid and lymphoid malignancies (Supplementary Table 2). Genomic DNA was isolated from tumor blasts from bone marrow samples with the QIAamp DNA Blood Kit (Qiagen GmbH, Hilden, Germany). Quality verified DNA extracted from tumor cells of patients was diluted to 5 ng/μL and prepared for library construction with the Ion AmpliSeq Library Kit 2.0 (Applied Biosystems, Foster City, CA, USA). The blast percentage of the samples ranged from 54.4 to 98.0%, with a median of 82.8% (Supplementary Table 1). The concentration of library was measured by Qubit 3.0. All consumable items and reagents used in Library pooling, Emulsion PCR and Enrichment of template were provided in Ion PGM Hi-Q View OT2 Kit and used according to the manufacturer’s instructions. The next-generation sequencing was conducted in the Ion torrent PGM (Applied Biosystems, Foster City, CA, USA) with Ion 318 chip. The procedure of transferring raw signal to base and reads alignment were conducted in the local server by default settings. The VCF (Variant Call Format) files containing the raw mutation information was uploaded to Ion Reporter server for further analysis. After getting MAF (Mutation Annotation Format) files, we filtered mutations by conditions: (1) MAF (Minor Allele Frequency) value < 0.01; (2) Mutation reads number > 10; (3) Coding region mutation; (4) Nonsynonymous mutation; (5) Not recorded in our false positive mutation database; (6) If the frequency was above 1% in any study of TopMed (freeze 8), gnomAD (v2.1.1), ExAC (v.0.3.1), The PAGE Study (phase II), 3.5KJPNv2, 1000Genomes (phase 3), KOREAN population from KRGDB (v1) and Korean Genome Project (phase I), and detected variant allele frequency was in 0.4–0.6 or 0.9–1.0, the mutation would be annotated as germline SNP and removed. IGV^32^ software (v2.6.3) was used to screening all mutations in BAM and filtering typical false positive mutations caused by targeted sequencing primers as described in McCall et al.’s work^33^. CEBPA, NPM1 and FLT3 were additionally sequenced by Sanger sequencing since of low coverage in NGS. Copy number variations (CNV) were calculated using CODEX2 (v1.3.0)^34^. Mutation burden was calculated by (aberrant CNV number + mutation number)/panel size (Mb).

### Statistical analysis

The baseline characteristics were compared by independent sample *t* test, Chi square test, or non-parameter test when appropriate. OS was measured from the date of diagnosis to the date of last follow-up or death from any cause. For progression-free survival (PFS) calculation, the uncensored event was relapse of leukemia and the censoring times reflected the end of follow up without an event or lost to follow up. The Kaplan–Meier survival analysis method was used to evaluate the prognosis determinants. The estimated survival values in different subsets were compared using the log-rank test and p < 0.05 were considered statistically significant. All analyses were performed using SPSS 21.0 (SPSS, Chicago, IL, USA).

### Ethics approval and consent to participate

The study design was approved by the Ethics Committee of Tongji Hospital, Tongji Medical College, Huazhong University of Science and Technology according to the Declaration of Helsinki and all patients gave written informed consents to participate in this study.

### Consent for publication

All authors are aware of and agree to this submission.

## Supplementary Information

Supplementary Tables.

## Data Availability

The raw data of next-generation sequencing are available at SRA (the sequence read archive) database of NCBI website (Bioproject: PRJNA646819).

## Abbreviations

AL: Acute leukemia
ALAL: Acute leukemia with ambiguous lineage
ALL: Acute lymphoid leukemia
AML: Acute myeloid leukemia
AUL: Acute undifferentiated leukemia
BAL: Bi-phenotypic acute leukemia
CAR-T: Chimeric antigen receptor T-cell
CR: Complete remission
EGIL: European Group for the immunological characterization of leukemia
HSCT: Hematopoietic stem cell transplantation
MPAL: Mixed-phenotype acute leukemia
NGS: Next-generation sequencing
OS: Overall survival
PFS: Progression free survival
TKI: Tyrosine kinase inhibitors
