# Supplementary material for: The association of complex genetic background with the prognosis of acute leukemia with ambiguous lineage
Source: Sci Rep. 2021 Dec 21;11:24290. doi: 10.1038/s41598-021-03709-7 (PMC8692450; doi:10.1038/s41598-021-03709-7)
Supplement: Supplementary file 1 — Supplementary Tables. [file 41598_2021_3709_MOESM1_ESM.doc]

| **Table S1. The morphology, immunophenotype and therapy summary of the patients in this study** | | | | | | | | |
| --- | --- | --- | --- | --- | --- | --- | --- | --- |
| **Patient NO.** | **EGIL** | | **Immunophenotype** | **WHO 2016** | **FAB** | **Blast percentage** | **Therapy Summary** | **Induction schedule** |
| **1** | BAL | | B-M | MPAL, B-M, NOS | ALL | 73.5 | ALL-like | VDCLP |
| **2** | BAL | | B-M | MPAL，BCR/ABL+ | AUL | 89.5 | ALL-like | VDCLP |
| **3** | BAL | B-M | | MPAL, B-M, NOS | mixed | 82 | AML+ALL-like | VDCLP+Ara-c |
| **5** | AUL | | AUL | AUL | AUL | 38 | AML-like | DA |
| **7** | BAL | | T-M | T-ALL | ALL | 97.5 | ALL-like | VDCLP |
| **8** | BAL | | T-M | MPAL, T-M, NOS | inconclusive | 81.5 | / | / |
| **10** | Triple-lineage | | B-T-M | MPAL, T-B, NOS | inconclusive | 76 | / | / |
| **11** | BAL | | B-T | MPAL, T-B, NOS | ALL | 66.5 | / | / |
| **12** | BAL | | B-M | B-ALL | inconclusive | 84 | AML+ALL-like | VP16+AA |
| **13** | BAL | | B-M | MPAL, T-B, NOS | ALL | 84 | AML+ALL-like | VDCLP+Ara-c |
| **14** | BAL | | T-M | T-ALL | inconclusive | 92 | AML-like | CAG |
| **15** | BAL | | T-M | MPAL，BCR/ABL+ | AML(M4) | 13.5 | AML+ALL-like | VDCP+HA+TKI |
| **16** | BAL | | T-M | MPAL, T-M, NOS | AML (M2) | 75.6 | AML+ALL-like | VP16+DA |
| **17** | BAL | | B-M | MPAL, B-M, NOS | inconclusive | 96 | AML+ALL-like | VDCP+HA |
| **19** | BAL | | B-T | MPAL, T-B, NOS | ALL | 90 | ALL-like | VDCLP |
| **21** | BAL | | B-M | MPAL，BCR/ABL+ | inconclusive | 84 | AML+ALL-like | VP+IA+TKI |
| **22** | BAL | | T-M | T-ALL | ALL | 79.5 | AML+ALL-like | VDCLP |
| **23** | BAL | | T-M | T-ALL | ALL | 97 | ALL-like | HyperCVAD-A |
| **25** | BAL | | T-M | MPAL, T-M, NOS | ALL | 54.4 | AML-like | IA |
| **26** | BAL | | B-M | MPAL，BCR/ABL+ | ALL | 31 | AML+ALL-like | VP+IA+TKI |
| **27** | AUL | | AUL | AUL | AUL | 98 | / | / |
| **28** | BAL | | T-M | MPAL, T-M, NOS | inconclusive | 59 | AML+ALL-like | VP+IA |
| **29** | BAL | | B-M | B-ALL | inconclusive | 69 | AML+ALL-like | VDCLP+Ara-c |
| **30** | BAL | | T-M | MPAL, T-M, NOS | AML(M1/M5) | 94.5 | AML+ALL-like | DVCP+HA |
| **31** | BAL | | B-M | MPAL，BCR/ABL+ | inconclusive | 70.08 | ALL-like | VP+TKI |
| **32** | AUL | | AUL | AUL | inconclusive | 92 | AML+ALL-like | VP+IA |
| **33** | BAL | | B-M | MPAL, B-M, NOS | ALL | 79.61 | ALL-like | VDCLP |
| **34** | BAL | | T-M | T-ALL | ALL | 92.4 | AML+ALL-like | VDCLP+Ara-c |
| **35** | BAL | | T-M | MPAL, T-M, NOS | AML(M5) | 54.98 | AML+ALL-like | DA+VP |
| **36** | BAL | | B-M | MPAL，BCR/ABL+ | AML (M1) | 0.98 | AML-like | IA |
| **37** | BAL | | B-M | MPAL，BCR/ABL+ | inconclusive | 88 | AML+ALL-like | VDCLP+Ara-c+TKI |
| **38** | BAL | | B-M | MPAL, B-M, NOS | inconclusive | 93 | / | / |
| **39** | BAL | | T-M | T-ALL | ALL | 96 | AML+ALL-like | HyperCVAD-A |
| **40** | BAL | | B-M | unclassified | inconclusive | 47 | AML-like | IA |
| **41** | BAL | | B-M | MPAL, B-M, NOS | inconclusive | 77 | AML-like | IA |
| **42** | BAL | | T-M | MPAL, T-M, NOS | ALL | 98.5 | AML+ALL-like | DA+VP |
| **43** | BAL | | T-M | MPAL, T-M, NOS | inconclusive | 56 | AML+ALL-like | VDCLP+Ara-c |
| **44** | BAL | | T-M | MPAL, T-M, NOS | inconclusive | 90 | AML+ALL-like | VDCLP+Ara-c |
| **45** | AUL | | AUL | AUL | ALL | 91 | AML+ALL-like | DVCP+HA |
| EGIL The European Group for the immunological characterization of leukemia; BAL biphenotypic acute leukaemia; | | | | | |  |  |  |
| AUL acute undifferentiated leukemia; MPAL mixed-phenotype acute leukemia; | | | | | |  |  |  |

| **Table s2. Detail information of the 173-gene panel in next-generation sequencing platform** | | | | | | | | | |
| --- | --- | --- | --- | --- | --- | --- | --- | --- | --- |
| ABL1 | ACTB | ACTG1 | AKT1 | ALK | ALPP | ARID1A | ASXL1 | ASXL2 | ATM |
| ATP6AP1 | ATP6V1B2 | B2M | BCL11B | BCL2 | BCL6 | BCOR | BIRC3 | BRAF | BRCC3 |
| BTG1 | BTK | CALR | CARD11 | CASP10 | CBL | CCND1 | CCND2 | CCND3 | CCR4 |
| CCR7 | CD28 | CD58 | CD70 | CD79B | CDKN1B | CDKN2A | CEBPA | CNOT3 | CREBBP |
| CRLF2 | CSF1R | CSF3R | CTCF | CTNNB1 | CXCR4 | DDX3X | DDX41 | DIS3 | DNM2 |
| DNMT3A | DTX1 | DUSP2 | EED | EGFR | EGR1 | EGR2 | EP300 | ETNK1 | ETV6 |
| EZH2 | FAM46C | FAM5C | FAS | FAT4 | FBXW7 | FGFR3 | FLT3 | FOXO1 | FYN |
| GATA1 | GATA2 | GATA3 | GNA13 | GNAS | GNB1 | HIST1H1E | HNRNPA2B1 | ID3 | IDH1 |
| IDH2 | IGLL5 | IKBKB | IKZF3 | IL7R | IRF4 | JAK1 | JAK2 | JAK3 | KDM6A |
| KIT | KLF2 | KLHL6 | KMT2C | KMT2D | KRAS | MAP2K1 | MAP2K2 | MAPK1 | MED12 |
| MEF2B | MPL | MYC | MYD88 | NF1 | NFE2 | NFKBIE | NOTCH1 | NOTCH2 | NPM1 |
| NRAS | NT5C2 | PAX5 | PDGFRA | PHF6 | PIK3CA | PIK3R1 | PIM1 | PKD1L2 | PLCG1 |
| PLCG2 | POT1 | POU2AF1 | POU2F2 | PRDM1 | PRKCB | PTEN | PTPN1 | PTPN11 | RAD21 |
| RHOA | ROBO1 | RPL10 | RPS15 | RRAGC | RUNX1 | SAMHD1 | SETBP1 | SF1 | SF3A1 |
| SF3B1 | SGK1 | SH2B3 | SMARCA4 | SMC1A | SMC3 | SOCS1 | SRSF2 | STAG1 | STAG2 |
| STAT3 | STAT5B | STAT6 | TBL1XR1 | TCF3 | TERF2IP | TET2 | TLR2 | TMSB4X | TNFAIP3 |
| TNFRSF14 | TNFRSF1B | TP53 | U2AF1 | U2AF2 | UBE2A | USP7 | VAV1 | VMA21 | WHSC1 |
| WT1 | XPO1 | ZRSR2 |  |  |  |  |  |  |  |

| **Table S3 Clinical outcomes of patients according to different parameters for all BAL/MPAL patients** | | | | |  |
| --- | --- | --- | --- | --- | --- |
| **Parameter** | | **Total** | **CR** | **p-value*** | |
|  |  | **No. of patients** | **No. of patients (%)** |  |  |
| **Immunophenotype** |  | |  | 0.163 | |
| **B/M** | | 15 | 11 (73.3) |  | |
| **T/M** | | 16 | 8 (50.0) |  | |
| **B/T+B/T/M** | | 4 | 1 (25.0) |  | |
| **Complex karyotype** | |  |  | 0.833 | |
| **Yes** | | 8 | 5 (62.5) |  | |
| **No** | | 21 | 14 (66.7) |  | |
| **Gene mutation** | |  |  | 0.208 | |
| **Yes** | | 15 | 8 (53.3) |  | |
| **No** | | 2 | 2 (100) |  | |
| **Mutation complexity^&^** | |  |  | 0.006^§^ | |
| **Low** | | 5 | 5 (100) |  | |
| **Middle** | | 8 | 2 (25) |  | |
| **High** | | 4 | 1 (25.0) |  | |
| **Treatment** | |  |  | 0.410 | |
| **ALL-like** | | 7 | 6 (85.7) |  | |
| **AML-like** | | 6 | 3 (50.0) |  | |
| **AML+ALL-like** | | 21 | 12 (57.1) |  | |
| **Classification system** | |  |  | 0.911 | |
| **WHO** | | 30 | 16 (53.3) |  | |
| **EGIL-WHO** | | 9 | 5 (55.6) |  | |
| CR: complete remission; WHO: World Health Organization; | | | | | |
| EGIL: European Group for the Classiﬁcation of Acute Leukemia; | | | | | |
| ^&^ calculated by (aberrant CNV number + mutation number)/panel size (Mb); low ≤2；middle=3； high ≥4. | | | | | |
| * evaluated by chi square test, §for p-trend | | | | | |

| **Table S4. Conventional risk factors for patients in survival analysis stratified by TMB** | | | | |
| --- | --- | --- | --- | --- |
| **Characteristics** | **MBT-low** | **MBT-middle** | **MBT-high** | **p value^＊^** |
|  | **(n=5)** | **(n=6)** | **(n=3)** |  |
| **Sex, m/f** | 3/2 | 3/3 | 3/0 | 0.513 |
| **Age, y** | 39±8.7 | 44±16.1 | 34±12.3 | 0.360 |
| **WBC, ×10^9^/L** | 21.5 (6.9-54.1) | 3.0 (1.8-9.8) | 4 (-) | 0.236 |
| **Complex Karyotype, N (% of tested)** | 0 (0) | 2 (40) | 1 (50) | 0.318 |
| **Treatment, ALL/AML/ALL+AML** | 2/1/2 | 1/0/4 | 0/0/2 | 0.596 |
| TMB: tumor mutation burden; ^＊^were compared by two-sided Fisher exact tests or Wilcoxon rank sum test; | | | | |

| **Table S5 Clinical outcomes of all BAL/MPAL patients according to different parameters** | | | | |
| --- | --- | --- | --- | --- |
| **Parameter** | **PFS, day** | **p-value^#^** | **OS, day** | **p-value^#^** |
|  | **median** |  | **median** |  |
| **Immunophenotype** |  | 0.035 |  | 0.065 |
| **B/M** | 578 |  | 731 |  |
| **T/M** | 264 |  | 299 |  |
| **B/T+B/T/M** | 205 |  | 205 |  |
| **Complex karyotype** |  | 0.684 |  | 0.670 |
| **Yes** | 422 |  | 731 |  |
| **No** | 424 |  | 360 |  |
| **Gene mutation** |  | 0.067 |  | 0.214 |
| **Yes** | 338 |  | 731 |  |
| **No** | / |  | / |  |
| **Mutation complexity^&^** |  | 0.001 |  | 0.001 |
| **Low** | 651 |  | 720 |  |
| **Middle** | 338 |  | 381 |  |
| **High** | 187 |  | 187 |  |
| **Treatment** |  | 0.856 |  | 0.086 |
| **ALL-like** | 651 |  | 580 |  |
| **AML-like** | 424 |  | 424 |  |
| **AML+ALL-like** | 338 |  | 360 |  |
| **Classification system** |  | 0.016 |  | 0.016 |
| **WHO** | 422 |  | 731 |  |
| **EGIL-WHO** | 207 |  | 211 |  |
| PFS: progression-free survival; OS: overall survival; WHO: World Health Organization; | | | | |
| EGIL: European Group for the Classiﬁcation of Acute Leukemia; ^#^ evaluated by Log-rank test; | | | | |
| ^&^ calculated by (aberrant CNV number + mutation number)/panel size (Mb); low ≤2；middle=3； high ≥4. | | | | |

| **Table S6 Clinical outcomes of patients according to different parameters by WHO system** | | | | | | |
| --- | --- | --- | --- | --- | --- | --- |
| **Parameter** | **CR** | **p-value*** | **PFS, day** | **p-value^#^** | **OS， day** | **p-value^#^** |
|  | **patients （%）** |  | **median** |  | **median** |  |
| **Immunophenotype** |  | 0.177 |  | 0.003 |  | 0.049 |
| **B/M** | 9 (75.0) |  | 641 |  | 571 |  |
| **T/M** | 5 (50.0) |  | 338 |  | 360 |  |
| **B/T** | 1 (25.0) |  | 205 |  | 205 |  |
| **Complex karyotype** |  | 0.604 |  | 0.521 |  | 0.665 |
| **Yes** | 5 (71.4) |  | 578 |  | 731 |  |
| **No** | 9 (60.0) |  | 346 |  | 351 |  |
| **Gene mutation** |  | 0.215 |  | 0.083 |  | 0.213 |
| **Yes** | 7 (53.8) |  | 252 |  | 360 |  |
| **No** | 2 (100) |  | / |  | / |  |
| **Mutation complexity^&^** |  | 0.022 |  | 0.002 |  | 0.002 |
| **Low** | 5 (100) |  | 651 |  | 720 |  |
| **Middle** | 2 (33.3) |  | 295 |  | 355 |  |
| **High** | 0 (0) |  | / |  | / |  |
| **Treatment** |  | 0.168 |  | 0.947 |  | 0.084 |
| **ALL-like** | 5 (100) |  | 422 |  | 602 |  |
| **AML-like** | 2 (66.7) |  | / |  | / |  |
| **AML+ALL-like** | 8 (57.1) |  | 348 |  | 370 |  |
| CR: complete remission; PFS: progression-free survival; OS: overall survival; * evaluated by chi square test | | | | | | |
| WHO: World Health Organization;;^#^ evaluated by Log-rank test; | | | | | | |
| ^&^ calculated by (aberrant CNV number + mutation number)/panel size (Mb); low ≤2；middle=3； high ≥4. | | | | | | |
